# Poster 2014: A survey of respiratory physicians' opinion of SIT in asthma treatment in Wuhan of China

**DOI:** 10.1186/1939-4551-7-S1-P25

**Published:** 2014-02-03

**Authors:** Rongfei Zhu, Nan Huang, Guanghui Liu

**Affiliations:** 1 Tongji Hospital, Tongji Medical College, HUST, China

## Background

Specific Immunotherapy has been applicated in allergic asthma for many years, however, in China, it's indication for asthma is still under controversial. The aim of our study is to investigate the respiratory physicians' opinion of SIT in asthma treatment in Wuhan, China.

## Methods

A 10-item questionnaire was designed in our study, the data were collected by telephone interview.

## Results

252 respiratory physicians were enrolled and 224 effective questionnaire were included in analysis. Among the 224 physicians, 92.9% had heard of SIT before and 75.9% regard SIT as an effective treatment in allergic diseases. However, only 55.8% thought SIT was proved to be effective in asthma.43.8% of the physicians agreed with that SIT had a long-term efficacy, 23.7% knew that there were two ways to perform SIT in clinic practice. But only 6.3% had prescribed SIT in their practice.

## Conclusions

More than 50% of the respiratory physicians in Wuhan have a positive attitude to SIT and it's application in asthma treatment. However SIT is not widely used in asthma patients and the efficacy is still under controversial in physicians of Wuhan.

